# Spin-textures of medium-body boson systems with trapped spin-f cold atoms

**DOI:** 10.1038/s41598-022-19184-7

**Published:** 2022-09-13

**Authors:** Y. Z. He, C. G. Bao, Z. B. Li

**Affiliations:** School of Physics, Sun Yat-Sen University, Guangzhou, 510275 People’s Republic of China

**Keywords:** Bose-Einstein condensates, Quantum mechanics, Theoretical physics

## Abstract

The spin-textures of bound medium-body systems with spin-$\mathfrak {f}$ atoms ($\mathfrak {f}\ge 3$) have been studied. The Hamiltonian is assumed to be dominated by the two-body interaction favoring parallel spins. The system with particle number $N=8$ and $\mathfrak {f}=3$ is first chosen, and the Hamiltonian is exactly diagonalized by using Fock-states as basis-states, thereby all the eigenenergies and eigenstates are obtained and a detailed analysis is made. Then the cases with $N=13$ and $\mathfrak {f}=4$ are further studied. Since the total spin *S* is conserved, the eigenstates having the same *S* form an *S*-group. Let the lowest (highest) energy state of an *S*-group be called a bottom-state (top-state). We found that all the bottom-states are bipartite product states with constituent states describing fully polarized subsystems containing $N_1$ and $N_2$ ($\le N_1$) particles, respectively. For two bottom-states different in $N_2$, the one with a larger $N_2$ is higher. For two having the same $N_2$, the one with a smaller *S* is higher. Whereas all the top-states are found to be essentially a product state of the pairs, in each pair the two spins are coupled to $\lambda$ if the strength of the $\lambda$-channel is more repulsive than the others. For the states belonging to an *S*-group, the higher one would contain more pieces. As the energy goes up, larger pieces (those containing more than two particles) will disappear.

## Introduction

It is well known that few-body theories and many-body theories are important for understanding the physical systems with the particle number $N\le 4$ and the systems with a large *N* (e.g., condensed matter systems), respectively. Basic structures in few-body systems (e.g., some solid pairs and/or clusters)^1–5^ and some basic physical processes (e.g., resonance in 2-body scattering and Efimov effect in diffuse 3-body systems) are known to play an important role in many-body systems^6–9^. For example, the $\alpha$-particles (the nucleus of helium atom) model in nuclear theory^10^ and the Cooper-pairs in superconductivity^3^. We believe that our understanding of few-body systems would be in general helpful for understanding the complicated phenomena occurring in many-body systems from a more fundamental point. However, the particle number *N* involved in few-body systems is greatly different from that in many-body systems. The few-body theories aim at finding exact solutions of Schrödinger equation. Each of them contains a complete set of good quantum numbers, and every physical feature of the system can be extracted from the solution. Whereas each many-body theory is skillfully designed and aims at finding approximate solutions for explaining specific physical phenomena (e.g., for explaining the ground state properties). Not all the good quantum numbers are conserved in these approximate solutions. To check the applicability of a many-body solution, an auxiliary way is to study in detail how the related physical features would vary with the increase of *N*, namely, to clarify the effect of *N*. This can be (partially) realized via the study of the medium-body systems (e.g., 5$\le N\le 20$). The knowledge from these systems would play the role as a bridge to relate the few- and many-body physics. Thus, the study of medium-body systems is worthy. The present paper is dedicated to these systems which are less studied previously.

An important progress of technology is the realization of extremely low temperature, e.g., $T\le 100$pK^11^. For the Bose-Einstein condensates, when *T* is sufficiently low, the spatial degrees of freedom can be frozen and, for particles with nonzero spin $\mathfrak {f}$, only the spin-degrees of freedom are active^12^. Since the spin-dependent force is much weaker than the central force, the spin-textures are very fragile and highly sensitive to a change in environment, e.g., a change in particle number and/or an adjustment in the strengths of interaction. Therefore, the spin degrees of freedom dominate the dynamics of such sensitive systems at $T\simeq 0$. Thus, the understanding of the spin-textures is important. Note that there are many literatures dedicated to the study of spin-$\mathfrak {f}$ systems with $\mathfrak {f}=1$^13–19^ and $\mathfrak {f}=2$^18–21^ atoms, but very few with high-$\mathfrak {f}$ systems^22–26^. Since the dimension of the spin-space increases greatly with $\mathfrak {f}$, the physics involved in high-$\mathfrak {f}$ systems would be rich. The present paper is dedicated to the study of the spin-textures in high-$\mathfrak {f}$ systems ($\mathfrak {f}\ge 3$) which are less studied previously.

Note that the formation of pairs (a coupling of two particles with their momenta and/or spins reversing to each other) is an important phenomenon in many-body systems, e.g., in superconductivity and in the interacting boson model (IBM) of nuclei^2,3^. In a previous paper^27^ the role of singlet pairs in high-$\mathfrak {f}$ condensates has been studied. The singlet pairs arise from the part of 2-body interaction which causes the two interacting spins to be anti-parallel. Thus, we think that it would be interesting to see what happens if the interaction leads to parallel spins.

As a continuation of the study in Ref.^27^, the present paper is dedicated to the study of high-$\mathfrak {f}$ medium-body systems with the interaction favoring to parallel spins. $N=8$ and 13, and $\mathfrak {f}$=3 and 4 are chosen as examples for medium-body high-$\mathfrak {f}$ systems. The strength of interaction of the 2$\mathfrak {f}$-channel is assumed to be much more attractive than those of other channels. The associated Hamiltonian is exactly diagonalized to obtain the spectra and the whole set of eigenstates. A detailed analysis has been made to find out the similarities and differences existing among the eigenstates of a spectrum and among the spectra of different systems (different in *N* and/or $\mathfrak {f}$). The emphasis is placed on finding out some basic spin-textures and the modes of excitation.

## The entire spectrum of a bound cold system with eight spin-3 atoms and with interaction leading to spin-parallel pair

We first choose a trapped 8-body system with spin-3 cold atoms as the first example for medium-body systems. Since we aim at the spin-textures, the temperature is assumed to be sufficiently low that all the spatial degrees of freedom are frozen and all particles fall into the same spatial state $\phi$ which is most favorable to binding. Note that, when the spin-orbit interaction appears, the lowest spatial state would in general mix up with higher excited spatial states. How strong the mixing would be, depends on the energy gap. Our assumption on the freezing is equivalent to having an infinite gap. Thus, the mixing is impossible. Therefore, the effect of spin-orbit coupling has been completely suppressed and can be neglected. Then the spin-3 boson system is governed by the spin-dependent Hamiltonian

1 $$\begin{aligned} H_{\mathrm {spin}} = \sum _{i<j}V_{ij},\ \ \ V_{ij} = \sum _{\lambda }g_{\lambda }P_{\lambda }^{i,j}, \end{aligned}$$

where $V_{ij}$ is the interaction between particles *i* and *j*, $g_{\lambda }$ is the weighted strength of the $\lambda$-channel ($\lambda =0,2,4$, and 6). A factor $\int \phi ^4(r)\mathrm {d}\mathbf {r}$, which embodies the effect of spatial profile, has already been included in $g_{\lambda }$. $P_{\lambda }^{i,j}$ is the projector to the $\lambda$-channel. Let $\chi$ denote a pure single spin-state, $(\chi (i)\chi (j))_{\lambda,m}$ denote a two-body spin-state in which the spins of *i* and *j* are coupled to $\lambda$ with magnetic number *m*. Then $P_{\lambda }^{i,j}=\sum _m|(\chi (i)\chi (j))_{\lambda,m}\rangle \langle (\chi (i)\chi (j))_{\lambda,m}|$. We consider the case that $H_{\mathrm {spin}}$ is dominated by the interaction that favors the formation of spin-parallel pair, i.e., $g_6$ is considered to be much more negative than the other three strengths so that the two interacting spins favor being parallel. The other three are first assumed to be equal to each other. Then, the case that they are unequal will be further considered.

**Figure 1:**
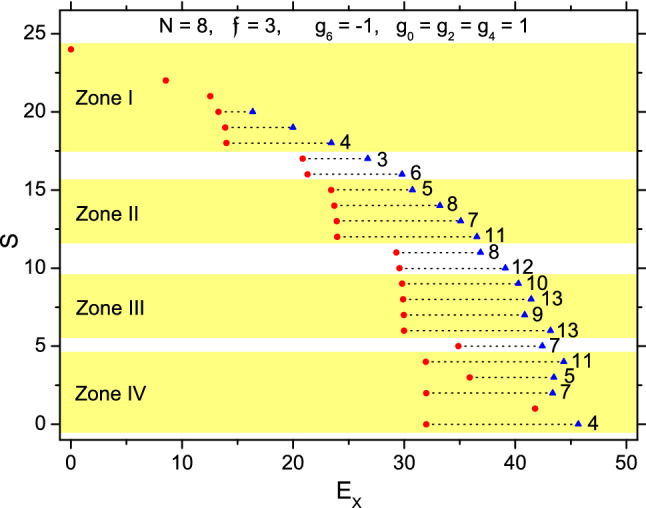
The complete spectrum of an 8-body system with spin-3 trapped cold atoms and with the Hamiltonian $H_{\parallel }$. The unit for energy is $(g_0-g_6)/2$. Totally there are 151 states (levels). The abscissa is for the excitation energies $E_X$ from 0 (the g.s.) to 45.68 (the highest energy state). The ordinate is for the total spin *S* of each state. The *S*-group of states are lying along a short horizontal dot-line. $E_X$ of the b-state and the t-state are marked by a red circle and a blue triangle, respectively. The multiplicity of an *S*-group is marked at the right of the dot-line if it $\ge 3$. When multiplicity$=1$, only a red circle is marked for the $E_X$ of the state. For an example, the group with $S=15$ contains 5 states and $E_X$ is distributed from 23.42 to 30.75.

Note that the spin-textures would not be changed if $\{g_{\lambda }\}$ as a whole is shifted with a common value and/or if the unit of energy is altered. Thus, for the first case, the Hamiltonian can be in general given as $g_6=-1$ and $g_0=g_2=g_4=1$, and is denoted as $H_{\parallel }$. By using the Fock-states as basis-states, $H_{\parallel }$ can be diagonalized to obtain the eigenenergies and eigenstates (refer to the Supplementary file Appendix). The total spin *S* and its *Z*-component *M* are good quantum numbers. A group of eigenstates having the same *S* form an *S*-group. Since magnetic field is not involved, it is sufficient to consider only $M=0$ states. The number of $M=0$ states included in an *S*-group is just the multiplicity of *S*. Let the lowest energy state of an *S*-group be called a bottom-state (b-state), while the highest a top-state (t-state). The spectrum of $H_{\parallel }$ with $N=8$ is plotted in Fig. 1, where the excitation energies $E_X$ of the b- and t- states of every *S*-group are marked. Thus, we can see how an *S*-group is distributed in the spectrum. When a state has a larger *S*, the spins are more likely to be parallel to each other. Therefore, when a negative $g_6$ is dominant, the *S*-group as a whole would be lower when *S* is larger as shown in Fig. 1. In particular, the ground state (g.s.) would have the largest $S=3N$, i.e., a fully polarized core.

Let a fully polarized subsystem be called a piece (p). Let the *i*-th eigenstate of an *S*-group ordered in uprising energy be denoted as $\Psi _{S,i}$. We found that $\Psi _{S,1}$, the b-state of each *S*-group, is mostly composed of two pieces containing $N_1$ and $N_2$ particles respectively ($N_1+N_2=N$). Let $[N_1]$ denote a piece with $N_1$ particles, $(N_1)_{S'}$ denote a subsystem where the $N_1$ spins are coupled to $S'$ (in particular, $(N_1)_{3N_1}\equiv [N_1]$). We found that, in Zone I marked in Fig. 1 with $24\ge S\ge 18$ (where the $S=23$ state is prohibited by symmetry), all the six b-states have $N_2=1$ and can be written as $\Psi _{S,1}=c([7]\chi )_S+\eta$, where $c\equiv \langle ([7]\chi )_S|\Psi _{S,1}\rangle \simeq 1$, $([7]\chi )_S$ represents the state in which the spin of a 7-body fully polarized subsystem (denoted by [7]) and the spin of a single atom are coupled to *S*, and $\eta$ is an *N*-body spin-state with an extremely small norm as shown in Table 1 (the case with $S=20$ is an exception). The associated excitation mode is a split of the fully polarized core into a $[N_1]$ piece, $N_1=N-1$, together with a single particle. Then the latter two, are coupled in various ways leading to the b-states with various *S*. For a state with a smaller *S*, the direction of the single spin would deviate more from that of the piece, therefore the probability of forming spin-parallel pairs is reduced. Accordingly, the state is higher in energy. In Zone II with $12\le S\le 15$, all the b-states have $N_2=2$ and very close to $([6][2])_S$. Thus, a pair of spin-parallel particles (i.e., [2]) have been extracted from the core. In Zone III with $6\le S\le 9$, all the b-states are very close to $([5][3])_S$, where a fully polarized triplet [3] has been extracted. In Zone IV with $0\le S\le 4$, all the b-states are very close to $([4][4])_S$. Thus, a higher excitation energy is needed to extract more particles from the core. The overlaps of the above suggested piece-piece states (p-p states) $([N_1][N_2])_S$ and the exact eigen-b-states $\Psi _{S,1}$ are listed in Table 1.

**Table 1:** The overlaps $$\langle ([N_1][N_2])_S|\Psi _{S,1}\rangle$$ ($$N_1+N_2$$ in short) and $$\langle ([N_1']\chi )_{3N_1'+1}[N_2])_S|\Psi _{S,1}\rangle$$ (($$N_1'+1)+N_2$$ in short) between the suggested p-p or p-1-p state and the exact eigen-b-state $$\Psi _{S,1}$$ of the Hamiltonian $$H_{\parallel }$$ with $$N=8$$ and $$\mathfrak {f}=3$$.

*S*	$7+1$	$(2+1)+5$		*S*	$6+2$	$(6+1)+1$	$(2+1)+5$
24	1			17	0.647	0.949	
22	1			16	0.379	0.975	
21	1			15	0.975		0.792
20		0.998		14	0.984		
19	0.998			13	0.999		
				12	1.000		

*S*	$5+3$	$(5+1)+2$	$(3+1)+4$	*S*	$4+4$	$(4+1)+3$	$(3+1)+4$
11	0.885	0.533	0.454	5		0.933	0.596
10	0.927	0.346	0.357	4	0.999		
9	0.995			3			0.989
8	0.997			2	0.999		
7	0.999			0	0.999		

Let *j* be an index to mark the order of all the eigenstates in up-rising energy. Thus, each state $\Psi _{S,i}$ can be equivalently denoted as $\Psi _j$. We found that the first excited state $\Psi _{j=2}$ is just the b-state $\Psi _{22,1}\simeq ([7]\chi )_{22}$, where a single spin has been extracted from the core and *S* is reduced. Let the mode leading to the transition $\Psi _1\rightarrow \Psi _2$ be called the cheapest excitation mode (CExM). Then, for a fully polarized core, the CExM is just to extract a single spin from the core together with a decrease of *S* from 3*N* to $3N-2$. Since the $S=3N-1$ state is prohibited by symmetry, the decrease in *S* implies that the single spin is no more lying along its previous direction but deviates from it as least as possible. We found from numerical diagonalization of $H_{\parallel }$ that the first excited state always has $S=3N-2$ for all *N*. Therefore, the idea of CExM can be generalized to any piece. Thus, when a p-p state is further excited, the lowest way is to excite a piece with CExM, namely, $[N_1]\rightarrow ([N_1']\chi )_{3N_1'+1}$, where $N_1'=N_1-1$. With this mode, a p-p state $([N_1][N_2])_S$ becomes a p-1-p state $(([N_1']\chi )_{3N_1'+1}[N_2])_S$.

From Table 1 we found that, while most b-states are a p-p state, a few of them (those lying between two zones, e.g., the group with $S=10$ is lying between Zone II and III) are a mixture of p-p and p-1-p as two basis-states. These basis-states are not orthogonal to each other. From the data given in the table together with the overlaps among the basis-states, the combined weight contributed by both basis-states can be known. For $\Psi _{5,1}$ as an example, the weight contributed by $(([4]\chi )_{13}[3])_5$ alone is $(0.933)^2$, by $(([3]\chi )_{10}[4])_5$ alone is $(0.596)^2$, while by both is 0.994. For $\Psi _{17,1}$, the weight contributed by $([6][2])_{17}$ alone is $(0.647)^2$, by $(([6]\chi )_{19}\chi )_{17}$ alone is $(0.949)^2$, while by both is 0.992. Thus, we conclude that all the b-states are composed of the p-p and p-1-p basis-states. An exception is the $S=1$ state. This state has multiplicity one, therefore its spin-texture is completely determined by symmetry and irrelevant to dynamics.

The second state of each *S*-group ($\Psi _{S,2}$) contains essentially three species (including $\chi$ and [2]), e.g.,

2 $$\begin{aligned} \Psi _{2,2}= & {} 0.972(([3]\chi )_{10}[4])_2+\eta \end{aligned}$$

3 $$\begin{aligned} \Psi _{4,2}= & {} 0.899(([4]\chi )_{13}[3])_4+0.290(([3]\chi )_{10}[4])_4+\eta \end{aligned}$$

4 $$\begin{aligned} \Psi _{18,2}= & {} 0.986(([2][2])_{10}[4])_{18}+\eta \end{aligned}$$

5 $$\begin{aligned} \Psi _{20,2}= & {} 0.872(([6]\chi )_{19}\chi )_{20}-0.355([6](2)_4)_{20}+\eta, \end{aligned}$$

where $\eta$ is a very small component ($\langle \eta |\eta \rangle =0.055, 0.029, 0.013$, and 0.002, respectively, for the above four cases). In these second states, we found again the further excitation of a piece via the CExM.

Furthermore, from Fig. 1 and Table 1 we found that $\Psi _{j=3}=\Psi _{21,1}=([7]\chi )_{21}$. Thus, the second CExM of a piece is to extract a particle together with a reduction of *S* by 3, i.e., $[N_1]\rightarrow ([N_1']\chi )_{3N_1'}$, where $N_1'=N_1-1$. In general, when the core splits up into more pieces and the relative orientations of the piece-spins are more deviated from each other, the associated excitation energy would be higher. As an example, we consider the split of [6] via CExM, i.e., $[6]\rightarrow ([5]\chi )_{15}$. With this mode, the p-p state $([6][2])_S$ would split further and becomes $(([5]\chi )_{15}[2])_S\equiv \Phi _{5-1-2,S}$. We found that there are totally seven $\Psi _{S,i}$ having $|\langle \Phi _{5-1-2,S}|\Psi _{S,i}\rangle |>0.8$ as listed in Table 2. These states with the CExM mode are all higher members of an *S*-group.

**Table 2:** All the eigenstates $\Psi _{S,i}$ of $H_{\parallel }$ with $N=8$ and $\mathfrak {f}=3$ having the overlap $|\langle (([5]\chi )_{15}[2])_S|\Psi _{S,i}\rangle |\equiv c>0.8$ are listed in the table.

*S*	*i*	*c*
10	4	0.926
11	3	0.861
14	5	0.891
17	3	0.954
18	3	0.876
19	2	0.958
20	2	0.828

When the energy goes higher, larger pieces $[N_1]$ with $N_1\ge 3$ will vanish gradually. Instead, more [2] and $(2)_{\lambda \ne 6}$ pairs will emerge. We are not going to the details of every excited states, but concentrate at the product states of the pairs. Let $\Phi _{S_A,S_B,S}\equiv (([2][2])_{S_A}([2][2])_{S_B})_S$. There are totally four eigenstates have the overlap $|\langle \Phi _{S_A,S_B,S}|\Psi _{S,i}\rangle |>0.96$. They are $|\langle \Phi _{10,8,3}|\Psi _{3,5}\rangle |=0.965$, $|\langle \Phi _{4,2,6}|\Psi _{6,2}\rangle |=0.995$, $|\langle \Phi _{10,4,13}|\Psi _{13,2}\rangle |=0.965$, and $|\langle \Phi _{10,8,17}|\Psi _{17,3}\rangle |=0.981$.

Note that, if $g_{\lambda _X}$ is most repulsive, the highest energy states would be dominated by the $(2)_{\lambda _X}$ pairs. For $H_{\parallel }$, there are three strengths equal to each other, i.e., $g_0=g_2=g_4$. In this case, we found that the highest energy state is

6 $$\begin{aligned} \Psi _{j=151}=\Psi _{0,4}=0.997([(2)_4(2)_4]_4[(2)_4(2)_4]_4)_0+\eta . \end{aligned}$$

Thus the $(2)_4$ pair is most important. The very high $S=1$ state is

7 $$\begin{aligned} \Psi _{j=142}=\Psi _{1,1}=([(2)_4(2)_4]_4[(2)_4(2)_2]_3)_1, \end{aligned}$$

where the $(2)_2$ pair emerges. Incidentally, the study of the highest energy states is meaningful because they would become the lowest energy states when the strengths reverse their signs.

**Figure 2:**
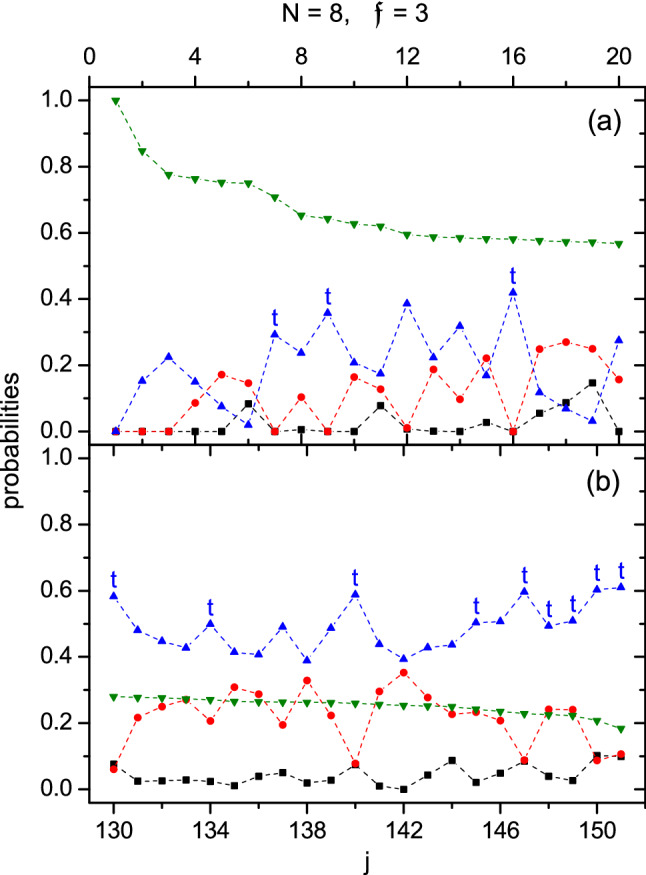
$\mathfrak {P}_{\lambda }$ against *j* for the case of $H_{\parallel }$ with $N=8$ and $\mathfrak {f}=3$. Probabilities, (**a**) for the states with $j\le 20$, (**b**) for $j\ge 131$. The symbols in black square, red circle, blue triangle, and olive reverse-triangle are for $\lambda =0,2,4$, and 6, respectively. All the t-states (the highest one of an *S*-group) are marked with a ’t’ (except those with multiplicity one, which happens when $j=1,2,3$, and 142).

Let $\mathfrak {P}_{\lambda }\equiv \langle \Psi |P_{\lambda }^{1,2}|\Psi \rangle$ be the probability of two particles in $\Psi$ forming a $(2)_{\lambda }$ pair. These probabilities against the index *j* are plotted in Fig. 2. Figure 2a is for $j=1\rightarrow 20$, where $\mathfrak {P}_6$ is much larger implying the importance of the fully polarized pieces, while $\mathfrak {P}_0$ is much smaller implying that the appearance of $(2)_0$ pairs is least probable. In average, $\mathfrak {P}_4>\mathfrak {P}_2$. The states with $j=7, 9$, and 16 are t-states with $S=20, 19$, and 18 (in Zone I of Fig. 1), respectively. Note that $\mathfrak {P}_4$ will arrive at a peak at the t-states. Therefore, for $H_{\parallel }$, the way to maximize the energy under the conservation of *S* is to increase the $(2)_4$ pairs. Figure 2b is for the highest 21 states with $j=130\rightarrow 151$, where $\mathfrak {P}_6$ is remarkably reduced implying the vanish of the polarized pieces, instead, $\mathfrak {P}_4$ becomes the largest and is also peaked at the t-states. This fact demonstrates again the domination of the $(2)_4$ pairs in high energy states (note that the highest energy state $\Psi _{j=151}$ is completely composed of the $(2)_4$ pairs). In any cases, $\mathfrak {P}_0$ remains to be small. Incidentally, the $(2)_0$ pairs would become dominant in high states when $g_0$ is most repulsive. This can be shown in the reverse of the spectrum given in Ref.^27^.

## The entire spectrum with $N=13$

**Figure 3:**
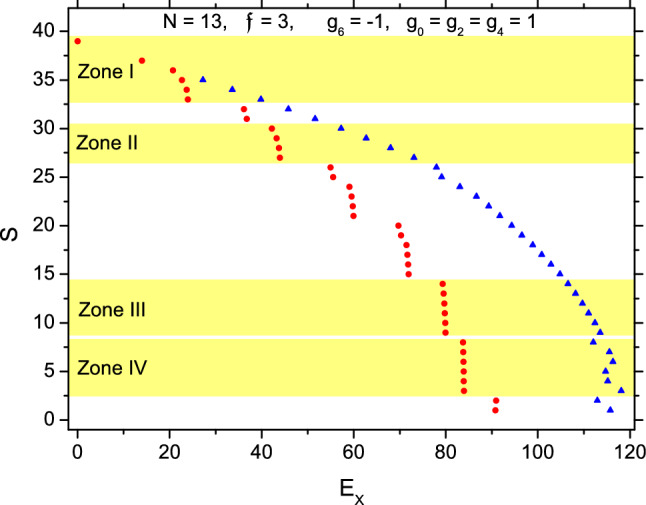
The spectrum of $H_{\parallel }$ with $N=13$ and $\mathfrak {f}=3$. Totally there are 920 levels, but only the excitation energies of the b- and t-states of every *S*-group are marked (refer to Fig. 1).

As the next example for medium-body systems, the whole spectrum of $H_{\parallel }$ with $N=13$ is plotted in Fig. 3.

**Figure 4:**
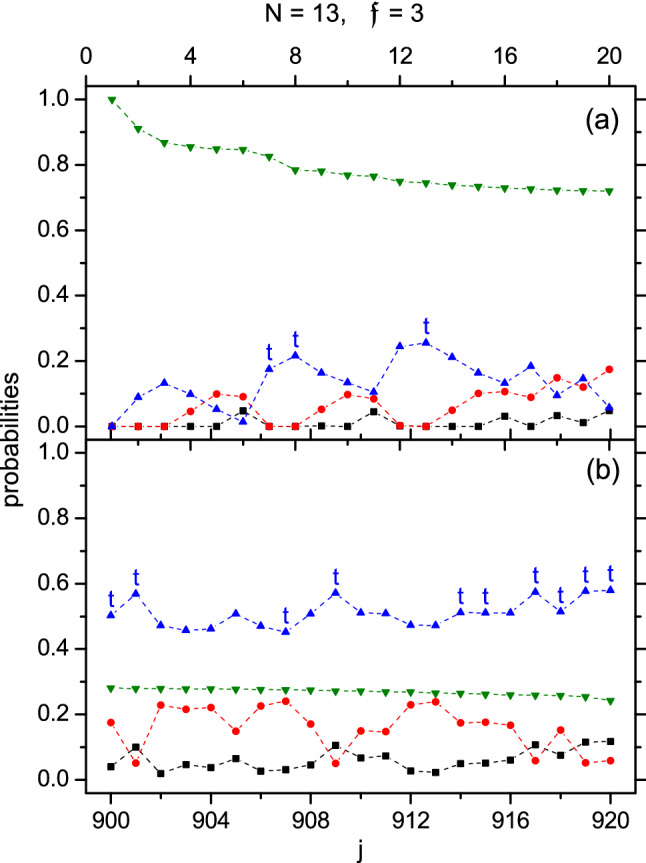
$\mathfrak {P}_{\lambda }$ against *j* for the case of $H_{\parallel }$ with $N=13$ and $\mathfrak {f}=3$ (refer to Fig. 2).

We found that the spectra of $N=13$ and $N=8$ systems are qualitatively similar. The p-p states $([N-1]\chi )_S$ recovers in the Zone I of Fig. 3, where $\Psi _{S,1}=c([12]\chi )_S+\eta$, $33\le S\le 39$ ($S=38$ is prohibited by symmetry), *c* is ranged from 0.998 to 1.000 (except that $\Psi _{35,1}$ has $c=0.966$). In particular, $\Psi _{j=2}=\Psi _{3N-2,1}\simeq ([N-1]\chi )_{3N-2}$, i.e., it is excited with the CExM, while $\Psi _{j=3}=\Psi _{3N-3,1}\simeq ([N-1]\chi )_{3(N-1)}$, i.e., excited with the second CExM. In Zone II of Fig. 3, the b-states $\Psi _{S,1}=c([N-2][2])_S+\eta$, $27\le S\le 30$, and *c* is ranged from 0.949 (when $S=30$) to 1. Thus, two spins have been extracted (i.e., $N_2=2$) as before. When *S* decreases further, more spins would be extracted. For example, in Zone III with $9\le S\le 14$, $N_2=5$ and $\Psi _{S,1}=c([8][5])_S+\eta$ with *c* being ranged from 0.977 (when $S=14$) to 1.000. In Zone IV, $N_2=6$, $3\le S\le 8$, and $\Psi _{S,1}=c([7][6])_S+\eta$, where *c* is from 0.998 (when $S=8$) to 1.000. In the above cases $\eta =(1-c^2)^{1/2}$ is so small that the split of the system into two fully polarized pieces is confirmed.

The probabilities $\{\mathfrak {P}_{\lambda }\}$ are plotted in Fig. 4. The t-states are marked with a ’t’ as before (The $j=1,2$, and 3 states are also t-states. Their multiplicity is one, therefore they are not marked). We found that Fig. 4 is qualitatively similar to Fig. 2. For example, $\mathfrak {P}_6$ ($\mathfrak {P}_4$) is more important in Fig. 4a (b), and $\mathfrak {P}_4$ is mostly peaked at the t-states.

## The spectra of the Hamiltonians deviated from $H_{\parallel }$

**Figure 5:**
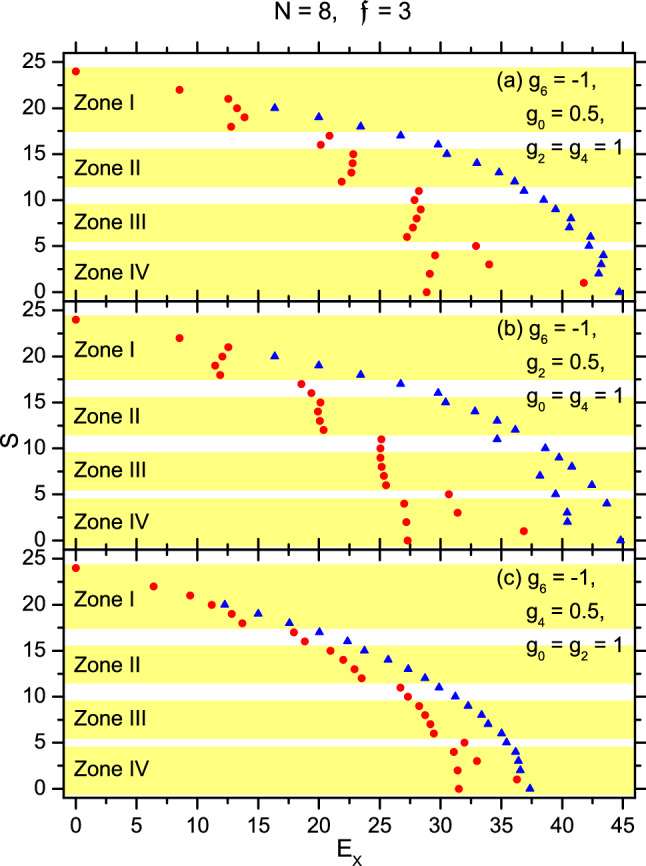
The spectra of an 8-body system with spin-3 trapped cold atoms. (**a**), (**b**), and (**c**) are for the Hamiltonian $H_{(i)}$, $H_{(ii)}$, and $H_{(iii)}$ respectively.

To evaluate the effect caused by a deviation from $H_{\parallel }$, we consider three cases (i) $g_0=0.5$, (ii) $g_2=0.5$, and (iii) $g_4=0.5$, while $g_6=-1$ remains unchanged and the other two strengths not mentioned in each above case have $g_{\lambda }=g_{\lambda '}=1$. The associated three deviated Hamiltonians are denoted as $H_{(i)}$, $H_{(ii)}$, and $H_{(iii)}$, respectively. Their spectra with $N=8$ are given in Fig. 5a, b, and c to be compared with Fig. 1. These figures are qualitatively similar to each other. For example, $E_X$ of the b-state varies interruptedly when *S* decreases and crosses over some critical values, these values are the same for $H_{\parallel }$ and $\{H_{(z)}\}$ ($z=i,ii$, and *iii*). Accordingly, the *S*-groups can be similarly divided into zones (e.g., the groups with $S=12$ to 15 are contained in Zone II in Fig. 1 and also in Fig. 5a, b, and c). The interruption implies a remarkable difference in spin-texture during the cross-over. For example, in the same zone of the four figures, the values $N_2$ specifying the p-p states $([N-N_2][N_2])_S$ are the same.

To reveal the similarity among the b-states with these different Hamiltonians, the overlaps $\langle ([7]\chi )_S|\Psi _{S,i}\rangle$ are given in Table 3 as an example, where all the b-states belong to Zone I with $24\ge S\ge 18$ ($S\ne 23$). The data demonstrate that all these states are dominated by the same basis-state $([7]\chi )_S$, thus they are nearly unchanged under the change $H_{\parallel }\rightarrow \{H_{(z)}\}$.

**Table 3:** The overlaps $\langle ([7]\chi )_S|\Psi _{S,1}\rangle$ (denoted as 7+1 in short) of $H_{\parallel }$ with $N=8$ and $\mathfrak {f}=3$, where $\Psi _{S,1}$ is an eigen-b-state of $H_{(z)}$ ($z=i$, *ii*, and *iii*) lying in Zone I.

*S*	$H_{\parallel }$	$H_{(i)}$	$H_{(ii)}$	$H_{(iii)}$
24	1	1	1	1
22	1	1	1	1
21	1	1	1	1
20	0.955	0.955	0.955	0.955
19	0.998	0.998	0.998	0.998
18	1.000	0.997	0.998	0.996

However, quantitatively, $E_X$ of the b-states given in Figs. 1 and 5 are slightly different. Note that, for $H_{\mathrm {spin}}$, the energy *E* of a spin-state $\Psi$ is

8 $$\begin{aligned} E = \langle \Psi |H_{\mathrm {spin}}|\Psi \rangle = \sum _{\lambda,i<j} g_{\lambda } \langle \Psi |P_{\lambda }^{i,j}|\Psi \rangle = \sum _{\lambda } g_{\lambda } \mathfrak {P}_{\lambda }. \end{aligned}$$

Thus, for the b-states in Zone I, the slight difference arises from the probabilities $\{\mathfrak {P}_{\lambda }\}$ inherent in the basis-state $([7]\chi )_S$, which is completely determined by symmetry as shown in Table 4.

**Table 4:** The set $\{\mathfrak {P}_{\lambda }\}$ of the p-p states $([7]\chi )_S$ of $H_{\parallel }$ with $N=8$ and $\mathfrak {f}=3$ different in *S*. In all these states, due to the fully polarized 7-body core, $\mathfrak {P}_6$ is dominant. However, when *S* decreases more from $3\times N=24$, the direction of the single spin deviates more from that of the fully polarized core, and therefore leads to a smaller $\mathfrak {P}_6$.

*S*	$\mathfrak {P}_0$	$\mathfrak {P}_2$	$\mathfrak {P}_4$	$\mathfrak {P}_6$
24	0	0	0	1
22	0	0	0.153	0.847
21	0	0	0.224	0.776
20	0	0.079	0.163	0.758
19	0	0.171	0.077	0.752
18	0.083	0.147	0.020	0.750

From this table, we know that, among the six $([7]\chi )_S$ states different in *S*, only the one with $S=18$ contains the $(2)_0$ pairs. Therefore, only this state would be benefited from the reduction of $g_0$. This explains why only the red circle for $\Psi _{18,1}$ is shifted to the left in Fig. 5a but not in Fig. 1, 5b, and 5c. In Fig.5b, $g_2$ is reduced. From the table we also know that the three states with $S=18,19$, and 20 contain the $(2)_2$ pairs, thus they would be benefited, in particular, the $S=19$ state would be benefited more. This is shown in Fig. 5b. In Fig. 5c $g_4$ is reduced. The energies of all the five states with $18\le S\le 22$ would be thereby reduced (that of the $S=21$ state would be reduced more) as shown in Fig. 5c.

Examples for the textures of high-lying b-states are shown in Table 5, where the overlaps $\langle ([5][3])_S|\Psi _{S,1}\rangle$ and $\langle ([4][4])_S|\Psi _{S,1}\rangle$ are shown.

**Table 5:** The overlaps $\langle ([5][3])_S|\Psi _{S,1}\rangle$ (denoted as $5-3$) and $\langle ([4][4])_S|\Psi _{S,1}\rangle$ ($4-4$), where $\{\Psi _{S,1}\}$ are the eigen-b-states of the associated Hamiltonians with $N=8$ and $\mathfrak {f}=3$, and they are lying in Zone III and IV. Refer to Table 2.

	$H_{\parallel }$	$H_{\parallel }$	$H_{(i)}$	$H_{(i)}$	$H_{(ii)}$	$H_{(ii)}$	$H_{(iii)}$	$H_{(iii)}$
*S*	$5-3$	$4-4$	$5-3$	$4-4$	$5-3$	$4-4$	$5-3$	$4-4$
9	0.995		0.977		0.984		0.971	
8	0.997		0.975		0.991		0.968	
7	0.999		0.996		0.995		0.995	
6	1.000		0.994		0.994		0.992	
4		0.999		0.991		0.994		0.989
2		1.000		0.995		0.994		0.993
0		1.000		0.993		0.993		0.990

It is impressive that all the overlaps given in Table 5 are also very close to one. Thus, during $H_{\parallel }\rightarrow H_{(z)}$, the domination of the $([N-N_2][N_2])_S$ textures recovers. The shift of $E_X$ occurring in Zone III and IV can be similarly explained as before.

To go beyond the b-states, we add a superscript in $\Psi _j$ as $\Psi _j^{H_{\parallel }}$ or $\Psi _j^{H_{(z)}}$ to specify the associated Hamiltonian. Let the overlaps $\{|\langle \Psi _j^{H_{\parallel }}|\Psi _{j'}^{H_{(z)}}\rangle |\}\equiv \{O_{j,j'}^{(z)}\}$. For a given $\Psi _j^{H_{\parallel }}$, among the 151 overlaps different in $j'$, the largest one is denoted as $O_j^{(z),\max }$. We found that, for the whole set of eigenstates of $H_{\parallel }$, 125 of them have $O_j^{(i),\max }>0.9$, thus each of them has an analogue in the set of eigenstates of $H_{(i)}$. The analogy implies that they are qualitatively unchanged during $H_{\parallel }\rightarrow H_{(i)}$. In particular, out of the 125, 41 of them have $O_j^{(i),\max }>0.99$ implying a high similarity. When $H_{(z)}=H_{(ii)}$, there are 108 eigenstates having $O_j^{(ii),\max }>0.9$ (wherein 33 of them have $O_j^{(ii),\max }>0.99$). When $H_{(z)}=H_{(iii)}$, there are 111 eigenstates having $O_j^{(iii),\max }>0.9$ (wherein 38 of them have $O_j^{(iii),\max }>0.99$). These data demonstrate that, for most eigenstates, the deviation caused by the deviated Hamiltonians is not large. Thus, the picture based on the pieces and the pairs $(2)_{\lambda }$ holds qualitatively when $H_{(z)}$ is not deviated from $H_{\parallel }$ seriously.

## The case with spin-4 atoms

**Figure 6:**
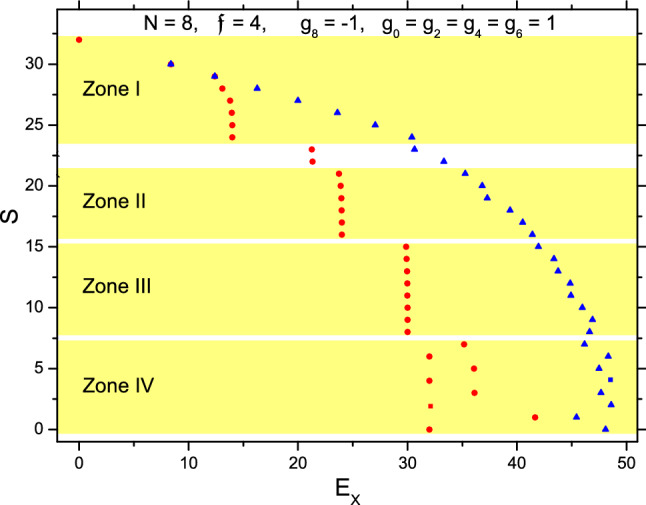
The spectrum of $H_{\parallel }$ with $N=8$ and $\mathfrak {f}=4$. Refer to Fig. 1.

For the systems with spin-4 atoms, $H_{\parallel }$ has $g_8=-1$ and $g_0=g_2=g_4=g_6=1$. After the numerical diagonalization of $H_{\parallel }$, the qualitative features of the eigenstates are found to be very similar to those with spin-3 atoms. In particular, the domination of the p-p texture $([N_1][N_2])_S$ in b-states, the further split of the pieces when energy goes higher, and the emergence of $(2)_{\lambda }$ pairs in high-lying states recover. The spectrum with $\mathfrak {f}=4$ and $N=8$ is plotted in Fig. 6, which is very similar to Fig. 1, e.g., the distribution against $E_X$ of each *S*-group and the division of the *S*-groups into zones. Note that in the Zone I of Fig. 1, $\Psi _{S,1}\simeq ([7]\chi )_S$, where the least value of *S* is $|7\mathfrak {f}-\mathfrak {f}|=18$. This is the same in Fig. 6 where the value is $|7\mathfrak {f}-\mathfrak {f}|=24$. Similarly, in Zone II, $\Psi _{S,1}\simeq ([6][2])_S$, therefore the least *S* is $|6\mathfrak {f}-2\mathfrak {f}|=12$ in Fig. 1 and 16 in Fig. 6. In Zone III, $\Psi _{S,1}\simeq ([5][3])_S$, therefore the least *S* is $|5\mathfrak {f}-3\mathfrak {f}|=6$ in Fig. 1 and 8 in Fig. 6.

## Summary and final remarks

Note that, for the high-$\mathfrak {f}$ systems with the pairwise interaction leading to spin anti-parallel pair, all the eigenstates are simply a product-state of the $(2)_0$ pairs together with certain number of unpaired particles^20,21,27^. Whereas in the present case with the interaction leading to spin parallel pair, we found that the g.s. is just a fully polarized core while the excitation mode is a split of the core, accordingly, the excited states are essentially composed of various pieces (fully polarized subsystems). The textures with more pieces and/or with the directions of the spins of the pieces deviated more from each other would lead to a higher energy. All the eigenstates are first classified into groups according to *S*. All the b-states of the *S*-groups have the p-p texture $([N_1][N_2])_S$ ($N=N_1+N_2$ and $N_1\ge N_2$). Thus the *S*-groups can be classified according to $N_2$. For the b-states having the same $N_2$, the one with a smaller *S* is higher. For the b-states with different $N_2$, the one with a larger $N_2$ is higher. When the excitation energy goes up, the pieces may split further, therefore the number of the pieces will increase and smaller pieces will emerge. In particular, those with particle number $N_p\ge 3$ would gradually vanish, and the eigenstates would be dominated by the pairs. For $H_{\parallel }$ with positive $g_0=g_2=g_4$, the highest energy states are found to be dominated by $(2)_4$ pairs. Whereas when $g_0\ne g_2\ne g_4$, it is believed that the $(2)_{\lambda _{o}}$ pairs would be dominant if $g_{\lambda _{o}}$ alone is most repulsive. This can be seen via the reverse of the spectrum given in Ref.^27^ where the $(2)_0$ pairs are dominant.

We have demonstrated numerically that the above picture holds when the Hamiltonian is not seriously deviated from $H_{\parallel }$. Besides, with the same approach of study, we found that the above picture holds also when $N\le 7$. It implies that some physical findings found in few-body systems could recover in medium-body systems. However, for many-body systems, a different approach of study is needed. How the above picture would be spoiled by increasing *N* remains to be further clarified. Although only the cases with spin-3 and spin-4 atoms are considered, we believe that, for $H_{\parallel }$, the above picture might hold for arbitrary integer $\mathfrak {f}\ge 1$. When $\mathfrak {f}$ is even and $g_{\mathfrak {f}}$ is most attractive (repulsive), the triplet with three spins coupled to zero might be dominant in the low-lying (high-lying) states. These assumptions deserve also to be further clarified.

## Supplementary Information

Supplementary Information.

## Data Availability

All data generated or analysed during this study are included in this published article (and its Supplementary Information files).

## References

[1] Bogoliubov NN (1947). On the theory of superfluidity. J. Phys. (USSR).

[2] Cooper LN (1956). Bound electron pairs in a degenerate Fermi gas. Phys. Rev..

[3] Bardeen J, Cooper LN, Schrieffer JR (1957). Theory of superconductivity. Phys. Rev..

[4] Arima A, Iachello F (1976). Interacting Boson model of collective states. I. The vibrational limit. Ann. Phys. (N.Y.).

[5] Tenart A, Hercé G, Bureik JP, Dareau A, Clément D (2021). Observation of pairs of atoms at opposite momenta in an equilibrium interacting Bose gas Nat. Phys..

[6] Efimov V (1970). Energy levels arising from resonant two-body forces in a three-body system. Phys. Lett. B.

[7] Nielsen E, Suno H, Esry BD (2002). Efimov resonances in atom-diatom scattering. Phys. Rev. A.

[8] Ferlaino F (2008). Collisions between tunable halo dimers: Exploring an elementary four-body process with identical bosons phys. Rev. Lett..

[9] Knoop S (2009). Observation of an Efimov-like trimer resonance in ultracold atom-dimer scattering. Nat. Phys..

[10] Ali S, Bodmer AR (1966). Phenomenological alpha-alpha potentials. Nucl. Phys. A..

[11] Deppner C (2021). Collective-mode enhanced matter-wave optics. Phys. Rev. Lett..

[12] Li ZB, Yao DX, Bao CG (2015). Spin-thermodynamics of ultra-cold spin-1 atoms. J. Low Temp. Phys..

[13] Stamper-Kurn DM (1998). Optical confinement of a Bose-Einstein condensate. Phys. Rev. Lett..

[14] Ho TL (1998). Spinor Bose condensates in optical traps. Phys. Rev. Lett..

[15] Ohmi T, Machida K (1998). Bose-Einstein condensation with internal degrees of freedom in alkali atom gases. J. Phys. Soc. Jpn..

[16] Law CK, Pu H, Bigelow NP (1998). Quantum spins mixing in spinor Bose-Einstein condensates. Phys. Rev. Lett..

[17] Elíasson O (2020). Spatial tomography of individual atoms in a quantum gas microscope. Phys. Rev. A.

[18] Koashi M, Ueda M (2000). Exact eigenstates and magnetic response of spin-1 and spin-2 Bose-Einstein condensates. Phys. Rev. Lett..

[19] Ott H (2016). Single atom detection in ultracold quantum gases: A review of current progress. Rep. Prog. Phys..

[20] Isacker P Van, Heinze S (2007). Bose-Einstein condensates of atoms with arbitrary spin. J. Phys. A Math. Theor..

[21] Kawaguchi Y, Ueda M (2012). Spinor Bose-Einstein condensates. Phys. Rep..

[22] Diener RB, Ho TL (2006). 52Cr spinor condensate: A biaxial or uniaxial spin nematic. Phys. Rev. Lett..

[23] Santos L, Pfau T (2006). Spin-3 chromium Bose-Einstein condensates. Phys. Rev. Lett..

[24] Makela H, Suominen K-A (2007). Ground states of spin-3 Bose-Einstein condensates for conserved magnetization. Phys. Rev. A.

[25] He L, Yi S (2009). Magnetic properties of a spin-3 chromium condensate. Phys. Rev. A.

[26] Kawaguchi Y, Ueda M (2011). Symmetry classification of spinor Bose-Einstein condensates. Phys. Rev. A.

[27] He YZ, Liu YM, Li ZB, Bao CG (2022). The band structure of the whole spectrum of an N-body cold system containing atoms with arbitrary integer spin and dominated by singlet pairing force. Phys. Scr..

