# Supplementary material for: Spin-textures of medium-body boson systems with trapped spin-f cold atoms
Source: Sci Rep. 2022 Sep 13;12:15357. doi: 10.1038/s41598-022-19184-7 (PMC9470676; doi:10.1038/s41598-022-19184-7)
Supplement: Supplementary file 1 — Supplementary Information. [file 41598_2022_19184_MOESM1_ESM.pdf]

Spin-textures of medium-body boson systems with trapped spin-f cold atoms

Y. Z. He[1], C. G. Bao[1], and Z. B. Li[1,*]

[1]School of Physics, Sun Yat-Sen University, Guangzhou, 510275, P. R. China
[*]Corresponding author: Z. B. Li, stslzb@mail.sysu.edu.cn

Appendix: Diagonalization of the Hamiltonian based on the Fock-states

For spin-$\mathfrak{f}$ boson systems, we introduce the Fock-states $|\alpha\rangle \equiv |N^{\alpha}_{\mathfrak{f}}, N^{\alpha}_{\mathfrak{f}-1}, \cdots, N^{\alpha}_{-\mathfrak{f}}\rangle$, where N^{α}_{μ} is the number of particles in magnetic component μ (-$\mathfrak{f} \le \mu \le \mathfrak{f}$), $\sum_{\mu} N^{\alpha}_{\mu} = N$ and $\sum_{\mu} \mu N^{\alpha}_{\mu} = M$, the total magnetization. They are adopted as basis-states for the diagonalization of $H_{\rm spin}$. The matrix element is

$$\begin{aligned}
\langle \alpha' | H_{\rm spin} | \alpha \rangle \;=\; & \frac{1}{2} \sum_{\mu'\nu'\mu\nu} \delta_{\mu'+\nu',\mu+\nu} \sum_{\lambda} g_{\lambda} C^{\lambda,\mu'+\nu'}_{\mathfrak{f}\mu';\mathfrak{f}\nu'} C^{\lambda,\mu+\nu}_{\mathfrak{f}\mu;\mathfrak{f}\nu} \\
& (\bar{\delta}_{\mu'\nu'} \bar{\delta}_{\mu\nu} \sqrt{N^{\alpha'}_{\mu'} N^{\alpha'}_{\nu'} N^{\alpha}_{\mu} N^{\alpha}_{\nu}}\, \delta_{[\alpha']_{\mu'\nu'};[\alpha]_{\mu\nu}} \\
& + \bar{\delta}_{\mu'\nu'} \delta_{\mu\nu} \sqrt{N^{\alpha'}_{\mu'} N^{\alpha'}_{\nu'} N^{\alpha}_{\mu} (N^{\alpha}_{\mu} - 1)}\, \delta_{[\alpha']_{\mu'\nu'};[\alpha]_{\mu\mu}} \\
& + \delta_{\mu'\nu'} \bar{\delta}_{\mu\nu} \sqrt{N^{\alpha'}_{\mu'} (N^{\alpha'}_{\mu'} - 1) N^{\alpha}_{\mu} N^{\alpha}_{\nu}}\, \delta_{[\alpha']_{\mu'\mu'};[\alpha]_{\mu\nu}} \\
& + \delta_{\mu'\nu'} \delta_{\mu\nu} \sqrt{N^{\alpha'}_{\mu'} (N^{\alpha'}_{\mu'} - 1) N^{\alpha}_{\mu} (N^{\alpha}_{\mu} - 1)}\, \delta_{[\alpha']_{\mu'\mu'};[\alpha]_{\mu\mu}}),
\end{aligned}$$

where $|\alpha'\rangle \equiv |N^{\alpha'}_{\mathfrak{f}}, N^{\alpha'}_{\mathfrak{f}-1}, \cdots\rangle$, $\delta_{\mu\nu} = 1$ or 0 (if $\mu = \nu$ or $\neq \nu$), $\bar{\delta}_{\mu\nu} = 1 - \delta_{\mu\nu}$, $[\alpha]$ denotes the set of $2\mathfrak{f}+1$ numbers $N^{\alpha}_{\mathfrak{f}}, \cdots, N^{\alpha}_{-\mathfrak{f}}$, $[\alpha]_{\mu\nu}$ ($\mu \neq \nu$) denotes the set $[\alpha]$ except that N^{α}_{μ} is changed to $N^{\alpha}_{\mu} - 1$ and N^{α}_{ν} is changed to $N^{\alpha}_{\nu} - 1$, $[\alpha]_{\mu\mu}$ denotes the set $[\alpha]$ except that N^{α}_{μ} is changed to $N^{\alpha}_{\mu} - 2$, $\delta_{[\beta];[\alpha]} = 1$ (if all the $2\mathfrak{f}+1$ numbers in $[\beta]$ are one-to-one identical to those in $[\alpha]$) or 0 (otherwise), the Clebsch-Gordan coefficients have been introduced. In the first (left) summation, each index runs from $-\mathfrak{f}$ to $\mathfrak{f}$. In the second summation, λ should be even and runs from 0 to $2\mathfrak{f}$. With these matrix elements, the diagonalization can be carried out numerically. Incidentally, 151 (920) basis-states are used when $\mathfrak{f} = 3$ and $N = 8$ (13).
